# A prospective study of the factors associated with life quality during medical internship

**DOI:** 10.1371/journal.pone.0220608

**Published:** 2019-08-12

**Authors:** Yu-Hsuan Lin, Hui-Yi Chen, Shih-Li Tsai, Li-Ren Chang, Pau-Chung Chen

**Affiliations:** 1 Institute of Population Health Sciences, National Health Research Institutes, Miaoli, Taiwan; 2 Department of Psychiatry, National Taiwan University Hospital, Taipei, Taiwan; 3 Department of Psychiatry, College of Medicine, National Taiwan University, Taipei, Taiwan; 4 Institute of Health Behaviors and Community Sciences, College of Public Health, National Taiwan University, Taipei, Taiwan; 5 Graduate Institute of Medical Education & Bioethics, National Taiwan University College of Medicine, Taipei, Taiwan; 6 Department of Medical Education, National Taiwan University Hospital, Taipei, Taiwan; 7 Institute of Environmental and Occupational Health Sciences, National Taiwan University College of Public Health, Taipei, Taiwan; 8 Department of Public Health, National Taiwan University College of Public Health, Taipei, Taiwan; 9 Department of Environmental and Occupational Medicine, National Taiwan University Hospital and National Taiwan University College of Medicine, Taipei, Taiwan; 10 Office of Occupational Safety and Health, National Taiwan University Hospital, Taipei, Taiwan; Universita degli Studi Europea di Roma, ITALY

## Abstract

**Background:**

Medical interns’ quality of life (QOL) are related to patient care quality, but the specific factors responsible for interns’ QOL have not been well studied. Herein we presented this nationwide, prospective study to examine the impact of working hours restrictions on the QOL among medical interns.

**Methods:**

The study recruited 295 medical interns (age: 25.3 ± 2.1, male: 68.1%) from all the 8 medical colleges in Taiwan during the 2012–2013 academic years. Subjects were assessed for QOL by brief version of the World Health Organization Quality of Life Assessment (WHOQOL-BREF) and the 9-item Patient Health Questionnaire (PHQ-9) before and every 3 months during their internship. We also investigated their demographic data, working hours, workload characteristics, such as specialties of internship rotation, acceptance of new patients after 24-hour, and no 24-hour off within 7 days, and self-reported patient related burnout. We used generalized estimation equation to delineate the change of WHOQOL-BREF and PHQ-9 scores during internship. We used multivariate regression analysis to examine the associated factors of QOL.

**Results:**

WHOQOL-BREF score significantly decreased during internship (baseline: 60.0 ± 9.7, 53.7 ± 9.3 at 3 months, then remained at 55 after 9 months). Acceptance of new patients after 24 hours of continuous duty (*β* = -2.089), no 24-hour off within 7 days (*β* = -1.748), score of patient related burnout (*β* = -2.50), and PHQ-9 depression score (*β* = -1.02) were associated with lower WHOQOL-BREF score. Working hours was not significantly associated with the QOL (*p* = .6268).

**Conclusions:**

Our findings revealed interns’ QOL significantly decreased during internship. Acceptance of new patients after 24-hour of continuous duty and patient related burnout predominantly impacted interns’ QOL and depression more than working hours did.

## Introduction

Internship per se is a well-controlled stress model, in which the onset of this major stressor was uniform. In addition, the subjects’ ages, lifestyles, and educational backgrounds are similar and they and they followed the same path of internship rotations with a similar working hours and workload. Therefore, the internship provides a unique human model to study the relationship between workload and mental health. In Taiwan, medical internship is a one-year training program in the seventh grade of medical school. Department of Higher Education, Ministry of Education regulated that medical interns should primarily care patient, such as managing patients’ complaints and acceptance of new patient under senior doctors’ supervision.

Medical interns typically work the greatest number of hours per week among all trainees who work in hospitals [1]. The Medical interns’ schedules consist of regular working hours and on-call duties. On-call duties among medical interns are characterized by sleep deprivation and mental stress, which they have never experienced before internship. Our previous studies showed that medical interns in Taiwan worked an average of 86.7 hours per week, and an average of 10 on-call duties per month with 33.5 consecutive working hours per duty [2–7]. The psychological and physiological impacts on medical interns during on-call duties included reduced cardiac sympathetic modulation [4], disrupted sleep stability [5], anxiety and depression [2], phantom vibration and ringing syndrome [3].

A number of studies have reported that training physicians experienced elevated depressive symptoms [8] and reduced quality of life (QOL) [9, 10], which may negatively impact on patient care quality [11]. In response to the recognition that fatigue from extended working hours, the Accreditation Council for Graduate Medical Education (ACGME) implemented the national regulation of duty hours for United States residents in 2003, and revised these regulations in 2011. The requirements included for a maximum of 80 weekly working hours, a 24-hour off within 7 days, and no new patients to be accepted after 24 hours of continuous duty. Since these regulations were implemented, few prospective studies [9, 10, 12, 13] have assessed the impact of working hours on interns’ mental health. One study [13] found working hours was associated with elevated depressive symptoms whereas another study [12] found that working hours had little contribution to depression. Moreover, previous studies [9, 12] suggested that suboptimal QOL, increased depression, and burnout, were still common during medical training course even under the intervention of working hours restrictions. Previous research focused on the role of working hours in elevated depressive symptoms and decreased QOL, but they have not further examined the influences of workload characteristics such as specialties of internship rotation, acceptance of new patients after 24-hour, and no 24-hour off within 7 days on interns’ mental health. However, these workloads do not necessarily lead to excessive working hours, and the impacts of workload characteristics may vary much on a par working hours. That is, in addition to working hours, workload characteristics may play a more important role in the development interns’ depressive symptoms and reduced QOL.

Physicians are prone to develop burnout in the medical training. Burnout is defined as a syndrome that includes emotional exhaustion, depersonalization, and a low level of personal accomplishment [14, 15]. Among high-achieving medical professionals, many evaluations of burnout focused on the either emotional exhaustion or depersonalization [10, 16, 17]. In addition, a recent study [18] demonstrated that the medical interns were most vulnerable to patient related burnout among training physicians. Another study [19] indicated the extents of patient related burnout was increasing throughout the internship year. Physicians’ QOL and burnout syndromes were associated with poor quality of patient care, medical errors, and reduced patient adherence to treatment plans [20].

Depression also linked to poor-quality patient care and increased medical errors. The prevalence of depression or depressive symptoms among resident physicians has been estimated at 28.8% [8] and among medical students at 14.3% [21]. In addition, medical interns’ on-call duties and long-term excessive working hours, associated with increased depression symptoms in this study, also accompanied by sleep disturbances [3]. Depressive symptoms and sleep disturbance, in particular insomnia, are significant risk factors of suicidal ideation and violent suicide attempts [22]. Depressed mood in a major depressive episode is more persistent and unable to anticipate happiness or pleasure, whereas the dysphoria in burnout is likely to correlate to with fluctuations in workload demands and accompanied by positive emotions and humor [23]. Although the conceptualization of professional burnout should be distinguished from depression [23], a longitudinal study showed strong correlations between burnout and depression both at baseline and at 2-year follow-up [24]. It is important that future longitudinal studies investigate the trajectory of professional burnout over the length of physicians’ careers.

We hypothesized that objective patient related workload, as well as self-reported patient related burnout, play a more essential role than working hours in medical interns’ QOL and depression. Thus, we examined the impacts of working hours, specialties of internship rotation, acceptance of new patients after 24-hour, no 24-hour off within 7 days and self-reported patient related burnout on interns’ QOL and depression. The specific aims of this study were (1) to use medical internship as a stress model to study subjects’ depressive symptoms and QOL before and after the onset of a major stressor, and (2) to identify the factors in the development of depression and impacts on QOL.

## Materials and methods

### Participants

We recruited 295 medical interns (201 males and 94 females, age 25.3±1.2 years) from 8 medical schools (NTU, NCKU, CGU, TMU, KMU, FJCU, CMU, and CSMU) in Taiwan (see S1 Table, for more details). All participants were the seventh grade of a medical college student, i.e. internship. Although all medical interns performed their internship at the different hospitals of their medical schools, they followed the same 12-month rotation, which was composed of four 3-month courses, which are (1) internal medicine, (2) surgery, (3) obstetrics/gynecology/pediatrics, and (4) other specialties. These 3-month courses were equal but the sequence of these courses might be different. The survey included demographics (age, gender and marital state), working hours and self-administered questionnaires: brief version of the World Health Organization Quality of Life Assessment (WHOQOL-BREF), 9-item Patient Health Questionnaire (PHQ-9) and occupational burnout inventory (OBI). Participants completed these questionnaires at months 3, 6, 9 and 12 of their internship year based on the fact that each course during the internship lasted 3 months and a baseline survey 1 to 2 months prior to beginning their internships. We administered the baseline survey 1 to 2 months prior to beginning their internships rather than the real and effective beginning because all of the medical schools had orientation and vestibule training for several days and some medical students had vacation for a couple of weeks before their internship. The study was carried out from May 2012 through to June 2013. All participants were given a detailed description of individual informed consent was obtained in written form. There were one or two medical interns in charge the data collection in each medical school, and they reminded interns who did not render back questionnaires once. There was missing data during this longitudinal follow-up because participants might forget or unwilling to fill in the questionnaire after these reminders. However, none medical interns took leave of absence in this study. This study was approved by the Institutional Review Board of National Taiwan University Hospital. The investigation was carried out in accordance with the latest version of the Declaration of Helsinki.

### Working hours estimation

During their internship, the participants had on-call duties, which they had never experienced before their internship. The total working hours included regular working hours and on-call duties. We estimated the total working hours per week. Consecutive working hours on duty comprised regular working hours, following on-call duty hours, and next regular working hours. For example, the regular working hours daily was from 08:00 to 17:00 (9 hours). On-call duty was from 17:00 to next 08:00 (15 hours), also followed by next 9 regular working hours. Thus, the consecutive working hours were 34 hours. This study also surveyed (1) if the medical interns accept new patients after 24 hours of continuous duty and (2) if they have a 24-hour off within 7 days.

### The brief version of the World Health Organization Quality of Life Assessment (WHOQOL-BREF)

The brief version of the World Health Organization Quality of Life instrument (WHOQOL-BREF) is a self-administered questionnaire that assesses quality of life (QOL) [25]. The WHOQOL instruments were developed worldwide, and has been culturally adapted into the Taiwan version, which is used extensively in Taiwan [26]. According to the psychometric criteria of the WHO, two (culturally relevant) national items were selected. The Taiwanese version WHOQOL-BREF included 28 items, consisting of 26 standard items from the original WHOQOL-BREF and two Taiwanese national items “Do you feel respected by others?” and “Are you usually able to get the things you like to eat?”. The 28-item WHOQOL-BREF classified into four domains: physical, psychological, social relationships, and environment. Each domain yields a score of 4 to 20, so that the WHOQOL-BREF total score ranges from 16 to 80. This study used total score of WHOQOL-BREF as primary outcome. Higher scores indicate better QOL. The psychometric analyses on the WHOQOL-BREF Taiwan version indicate that this questionnaire is reliable and valid [26].

### Patient Health Questionnaire (PHQ-9)

The PHQ-9 is widely used self-administered instruments for detecting depressive symptoms. For each of the 9 depressive symptoms, subjects indicated whether, during the past 2 weeks, the symptom had bothered them “not at all,” “several days,” “more than half the days,” or “nearly every day.” Each item yields a score of 0 to 3, so that the PHQ-9 total score ranges from 0 to 27. A score of 10 or greater on the PHQ-9 has a sensitivity of 93% and a specificity of 88% for the diagnosis of major depressive disorder [27]. Diagnostic validity of the Chinese version PHQ-9 is comparable with clinician-administered assessments [28].

### Patient related burnout

This study used client-related burnout subscale in the Chinese Occupational Burnout Inventory (OBI) and substitute “patients” for “clients” in each item because our previous study showed client-related burnout had an independent impact on the presence of phantom vibration and phantom ringing syndrome [29], which were prevalent hallucination during medical internship [3]. The “patient related burnout” subscale includes six items. Each items have five response categories. The responses are rescaled to a score of 0–100 (the values being 0–25–50–75–100). The patient related burnout subscale score was calculated by taking the mean of the items and ranges from 0–100, with higher scores indicating a greater degree of patient related burnout [30].

### Statistical analysis

For descriptive statistics, mean ± standard deviation (SD) for continuous variables such as age, working hours, and scores of patient related burnout were calculated. The number and frequency (%) of gender, specialties of internship rotation, interns who had working hours greater than 80 hours/week, acceptance of new patients after 24 hours of continuous duty, and no 24-hour off within 7 days were also reported. To find out effectors of the WHOQOL-BREF and PHQ-9 depression score, generalized estimation equation (GEE) was implemented to deal with the longitudinal data. GEE model was applied here as an alternative to traditional techniques such as repeated measure ANOVA that do not handle missing data and time-dependent covariates properly.

Effectors of changes in WHOQOL-BREF and PHQ-9 depression score during internship from baseline were examined with linear regression. The dependent variables were difference scores from baseline of PHQ-9 and WHOQOL-BREF respectively. Because the increased PHQ-9 scores and decreased WHOQOL-BREF scores from baseline both remained steady across 3, 6, 9 and 12 month periods during internship, we did not treat this time period as an independent variable. Instead, we examined the effects of specialties of these 3-month internship rotations. When skewed data distributions were taken into account, normalization was applied to data before the regression analysis was carried out because these raw data did not present normal distribution. The normalization was done using the following formula: (raw data—group mean)/ SD of group. Parameters which had P<0.100 in univariate analysis were then used for multivariate analysis, and a final model was determined after the procedure of backward selection. All statistics are two-sided and performed with SAS statistical software (version 9.2, SAS Institute Inc., Cary, NC). A *p* value < .05 was considered statistical significant.

## Results

Table 1 shows characteristics of 295 students from baseline to 3-months, 6-months, 9-months, and 12-months during the internship. The mean age of 25.3 ± 2.1 years, 68.3% were males, and most (99%) were single. There was no significant difference in specialties of internship rotation distribution during 3,6,9,12-months of the internship. The average weekly working hours prior to internship was 49.0 and significantly increased to 85.7–90.9 hours during their internship. There was no significant difference of the working hours at the 3, 6, 9, 12-month. In addition, 59.5%-70.4% interns worked more than 80 hours per week during internship. 10.8%-30.8% interns did not follow the rule of at least one day off within a week. 60.6% - 66.5% interns have to accept new patients during regular working hours after an overnight shift of 24-hour duration. The score of patient related burnout was 37.8 at baseline and increased to 45.8–46.3 during the all internship.

**Table 1:** Characteristics at baseline and follow-ups.

Variables	Before internship	3 months	6 months	9 months	12 months
**Age, years**	25.32±2.06	-	-	-	-
**Gender, males (%)**	200 (68.26)	-	-	-	-
**Specialties of internship rotation**					
Baseline	295 (100)	-	-	-	-
Internal medicine	-	74 (31.90)	63 (32.81)	37 (26.43)	44 (30.56)
Surgery	-	63 (27.16)	51 (26.56)	32 (22.86)	31 (21.53)
Pediatric, obstetrics and gynecology	-	53 (22.84)	41 (21.35)	40 (28.57)	29 (20.14)
Others	-	35 (15.09)	34 (17.71)	27 (19.29)	33 (22.92)
**Working hours per week**	49±26.66	90.86±19.47	85.96±20.11	85.70±28.64	85.73±20.94
> 80 hours/ week	26 (15.2)	133 (70.37)	91 (59.48)	75 (65.79)	81 (66.39)
**Acceptance of new patients after 24 hours of continuous duty**	107 (49.08)	151 (66.52)	115 (61.17)	90 (66.18)	86 (60.56)
**No 24-hour off within 7 days**	66 (27.85)	70 (30.84)	41 (21.81)	29 (21.80)	15 (10.79)
**Score of patient related burnout**	37.75±16.25	45.83±18.45	45.91±18.50	46.25±19.70	46.21±17.91

Categorical data were summarized as n (%) and continuous data were as mean±SD.

Medical interns who were lost follow-up had lower baseline WHOQOL-BREF scores (50.1±7.9 vs. 52.6±7.2, p = 0.006) and higher percentage of no 24-hour off within 7 days (25.9% vs. 22%, p = 0.044) than interns who completed the 12 months survey. There was no significant difference in age, working hours, baseline depression scores, patient burnout scores, or percentages of gender, acceptance of new patients after 24 hours of continuous duty.

### Depression

GEE model showed that the PHQ-9 depression score increased from 4.4 ± 4.0 at baseline to 7.1 ± 5.3 at 3 months and did not significantly change at the 3, 6, 9, 12-month of internship. Table 2 summarizes variables associated with depression score from baseline with follow-up by 3-months, 6-months, 9-months, and 12-months. The increased PHQ-9 depression score during the past 3 months were associated with working hours (*β* = 0.013), acceptance of new patients after 24-hour continuous duty (*β* = 0.904), and the score of patient related burnout (*β* = 0.496). All specialties of internship rotation were associated to increased depression scores from baseline with borderline *p* values (internal medicine: *β* = 0.919, *p* = 0.057; surgery: *β* = 1.048, p = 0.056; pediatric, obstetrics and gynecology: *β* = 0.851, *p* = 0.066), except other specialty (*β* = 0.953, *p* = 0.042).

**Table 2:** Effectors of self-rating depression score.

Variables	Multivairate analysis				
	B (95%CI)			*p*	
**Specialties of internship rotation**					
Baseline	Reference				
Internal medicine	0.919 (-0.028,1.866)			0.057	
Surgery	1.048 (-0.024,2.119)			0.056	
Pediatric, obstetrics and gynecology	0.851 (-0.056,1.758)			0.066	
Others	0.953 (0.035,1.872)			**0.042**	
**Working hours per week**	0.013 (0.001,0.025)			**0.041**	
**Acceptance of new patients after 24 hours of continuous duty**	0.904 (0.317,1.480)			**0.003**	
**No 24-hour off within 7 days**	0.828 (-0.019,1.675)			0.055	
**Score of patient related burnout**	0.496 (0.385,0.608)			**< .001**	

### Quality of life

GEE model showed that the WHOQOL-BREF score significantly decreased from 60.0 ± 9.7 at baseline to 53.9 ± 9.2 at 3 months and did not significantly change at the 3, 6, 9, 12-month. Table 3 summarizes variables associated with WHOQOL-BREF score from baseline with follow-up by 3-months, 6-months, 9-months, and 12-months. WHOQOL-BREF score decreased along with interns in the course of internal medicine (*β* = -2.522), surgery (*β* = -1.909), acceptance of new patients after 24 hours of continuous duty (*β* = -2.089), 24-hour off within 7 days (*β* = -1.748), score of patient related burnout (*β* = -0.502), and PHQ-9 depression score (*β* = -1.015). Working hours did not significantly decrease QOL.

**Table 3:** Effectors of WHOQOL-BREF.

Variables	Multivairate analysis	
	B (95%CI)	*p*
**Specialties of internship rotation**		
Baseline	Reference	
Internal medicine	-2.522 (-4.064,-0.981)	**0.001**
Surgery	-1.909 (-3.578,-0.240)	**0.025**
Pediatric, obstetrics and gynecology	-0.284 (-1.883,1.315)	0.728
Others	0.341 (-1.235,1.917)	0.672
**Working hours per week**	-0.005 (-0.024,0.014)	0.627
**Acceptance of new patients after 24 hours of continuous duty**	-2.089 (-3.140,-1.037)	**< .001**
**No 24-hour off within 7 days**	-1.748 (-2.928,-0.569)	**0.004**
**Score of patient related burnout**	-0.502 (-0.710,-0.294)	**< .001**
**Total of self-rating depression score**	-1.015 (-1.176,-0.854)	**< .001**

## Discussion

This longitudinal nationwide cohort study demonstrated that acceptance of new patients after 24-hour of continuous duty, and patient related burnout was associated with reduced interns’ QOL and elevated depressive symptoms more than working hours was. In addition, we identified the details of the characteristics of workload, including patient related burnout, if medical interns accept new patient after 24 hours of continuous duty and 24-hour off within 7 days. Furthermore, the unique internship rotation program in Taiwan provide evidence to identify the role of medical specialties in physicians’ QOL based on within-subject comparison. We study the changes of depressive symptoms and QOL during internship, especially after 3-months from the beginning because these stress-related conditions, such as an adjustment disorder with depressed mood typically developed within 3 months after the onset of the stressor and resolved within 6 months of the termination of the stressor [31].

### Quality of life

Interns’ subjective patient related burnout, whether medical interns accept new patient after 24 hours of continuous duty, was significantly associated with reduced QOL. Interns’ QOL was also associated with no 24-hour off within 7 days. These factors were proposed by Accreditation Council for Graduate Medical Education in 2003. However, to our best knowledge, these rules has not been investigated their relevance to QOL specifically in previous studies. Patient related burnout scores were reported by interns’ subjective experiences of a physical and psychological exhaustion in patient related workload, while acceptance of new patient after 24 hours of continuous duty was an objective measurement of patient related workload. With both subjective and objective measurements, our results revealed that patient related workload may leave medical interns susceptible to the reduced QOL and elevated depression. Furthermore, there was no significant correlation between weekly working hours and QOL during the internship, which was consistent with the results of a previous study for surgical interns [9].

Specialties was not a risk factor of depression among interns, but internal medicine and surgery were associated with lower QOL during internship. Although previous studies have assessed QOL and depression in physicians, few studies used the same subjects to compare the effect of the specialties on depression and QOL. The unique internship rotation program in Taiwan provided a rare model to explore the role of medical specialties in physicians’ depression and QOL based on within-subject comparison because each medical interns had rotated for four specialties: internal medicine, surgery, obstetrics/gynecology/pediatrics and other specialties throughout the course of the internship year.

We found that internal medicine and surgery were associated with lower QOL during internship, and these findings were consistent with the suboptimal QOL in surgical interns and internal medicine residents in previous studies [9, 12]. The internal medicine and surgery rotations especially might mire interns to difficulties to balance both personal life and work responsibilities despite controlling the work hours and scores of burnout. A similar result in a national study showed that one-third of surgical interns still perceived suboptimal balance between their personal and professional lives despite the duty hour reductions in 2011 [9]. Interns in their surgery rotation may be uniquely susceptible to have lower QOL due to the pressure of mastering both technical expertise and medical knowledge [9, 32–34]. A recent systematic review suggested that stressors commonly encountered by surgeons, such as family life, fear of failure, poor performance, hostile work environment, and comorbidities, may play significant roles in surgeon QOL [35]. Different from surgeons have to keep a high level of attention throughout their operation, medical interns in the internal medicine rotations attend to their patients’ complaints in a fragmentary fashion throughout the day [5, 6]. Medical knowledge may especially influence on their QOL during internal medicine rotations in the context of potentially stressful direct patient care activities [12].

### Depression

Depressive symptoms were increased within first 3 months and remained through the internship year. This trend was similar to previous research [2, 3, 7]. Interns’ depression was most associated with scores of patient related burnout and whether they had to accept new patient after 24 hours of continuous duty. In contrast, specialties of internship rotation did not play a major role in the increasing depressive symptoms. Therefore, interns’ increased depression resulted from their clinical workload and did not differ among specialties of rotation. Our study assessed the intern’s depressive symptoms by PHQ-9 which was used widely in most previous studies investigating medical intern’s depression. Using the same scale contributed to comparisons across studies. In our study, medical interns had PHQ-9 depression score of 4.4 and weekly working hours of 49.0 at baseline. PHQ-9 depression scores were also elevated to 6.24–7.15 during the internship with increased weekly working hours of 85.7–90.9. Similarly, in a previous large study [13] in the United States, their interns had PHQ-9 depression score of 2.4 before internship and their PHQ-9 depression score was increased to 6.26–6.7 and average weekly working hours were 64.89–68.70 within the internship. In our study, the average depression score of 4.4 ± 4.0 before internship and 6.24–7.15 during internship were at the level of subclinical depression, compared to the cutoff score of 10 in PHQ-9 [27]. These results was consistent with the findings of almost all large studies [2, 12, 13] that interns’ depression scores were at the level of mild depression and may not meet the DSM-5 criteria of ‘‘marked distress that is in excess of what would be expected given the nature of the stressor, or by significant impairment in social or occupational functioning”. Increased depression scores during internship did not meet the clinical meaning of worsening depression. It is conceivable that the increased symptoms of depression during the internship did not result in significant impairment in occupational functioning. Therefore, our findings suggested that the increased depressive symptoms during internship were similar to adjustment disorder with depressive mood rather than major depressive disorder [31]. In addition, only “other specialties” significantly increased depression scores. Among all internship rotation, only “other specialties” consisted three or more specialties, such as psychiatry, neurology, radiology, pathology, in this 3-month training course, whereas internal medicine, surgery and obstetrics/gynecology/pediatrics rotations had more homogeneousness in their 3-month courses. A qualitative study demonstrated the major stressors during medical internship including taking responsibilities, facing uncertainties and establishing relationship with their supervisors and nurses [36]. During the “other specialties” rotations, medical interns faced uncertainties more frequently and had to established more relationship with their supervisors and nurses. Thus, medical interns had to cope with more stressors in “other specialties” rotation, and had significant higher depression scores.

In the present study, there was no significant association between working hours and QOL after controlling other confounding factors in multivariate analysis. These findings suggested excessive working hours may result from several characteristics of workload. Acceptance of new patient after 24 hours of continuous duty, and 24-hour off within 7 days had greater influences on elevated depressive symptoms than working hours did. We also identified that patient related burnout and if medical interns accept new patient after 24 hours of continuous duty, were associated to QOL and depressive symptoms. Medical interns who accepted new patients after 24 hours of continuous duty had elevated depressive symptom score of 0.9, whereas those who have an additional 10 working hours per week (average additional 2 working hours per day) had relatively small increased score of 0.13 (0.013×10).

There are several methodological limitations that should be noted when interpreting our findings. First, there may be recall bias among interns when reporting depressive symptoms presented during each 3-month course; furthermore, our study lacks a more structured interview that is needed to confirm the depressive symptoms. Second, the heterogeneity among different specialties, sequence of rotations, and hospitals might confound the implications of our study. Some participants failed to follow-up at in this longitudinal study. However, the nationwide and longitudinal study design could still represent the country in general. Third, there was no control group to examine whether the changes of QOL and depression caused by the stress during internship. Fourth, it is possible that unmeasured variables were present, such as individual stressful life event and personality traits, which were well-known risk factors of depressive symptoms [37]. In addition, the quality of internship supervision might be an important factor in the onset of stress, the future studies should apply an evaluation form to have an index of the quality of supervision. Fifth, missing data from interns lost follow-up might influence our findings. We used generalized estimation equation (GEE) for statistical analysis. GEE assumed that “missing data are missing completely at random (MCAR)”. It is more stringent than other statistical models usually used to analysis longitudinal data [38, 39]. We further conducted Little’s MCAR test and the result showed the missing values in our data is MCAR (*χ*^*2*^ = 1269.970, *p* = .495). It revealed the loss of subjects did not impact the applicability of GEE. We compared the data of 55 medical interns completed all five visits follow-up and the present data of 295 medical interns (S2 and S3 Tables). These key findings were still partly valid and the *β* estimates were similar. However, the statistical significance (*p* value) dampened because of the substantial reduced sample size. Finally, we did not investigate interns’ QOL and depression after their internship because not all of the interns did not have to work. This setting limit the causal inferences interpretation of QOL and the associated within-Internship Factors.

In conclusion, acceptance of new patients after 24-hour of continuous duty and patient related burnout had more essential roles on medical intern’s depression and QOL than working hours did. These factors have been clearly linked to patient safety concerns. In addition to working hours regulation to medical internship, the internship supervision is needed to improve the quality of interns’ workload.

## Supporting information

S1 Table Characteristics for students from eight medical schools at baseline (*N* = 295). (DOCX)

S2 Table Effectors of self-rating depression score (*N* = 295 vs. *N* = 55). (DOCX)

S3 Table Effectors of WHOQOL-BREF (*N* = 295 vs. *N* = 55). (DOCX)
