# Supplementary material for: A prospective study of the factors associated with life quality during medical internship
Source: PLoS One. 2019 Aug 12;14(8):e0220608. doi: 10.1371/journal.pone.0220608 (PMC6690540; doi:10.1371/journal.pone.0220608)
Supplement: S1 Table — (DOCX) [file pone.0220608.s001.docx]

| **S3 Table.** Characteristics for students from eight medical schools at baseline (*N* = 295) | | | | | | | | | | |
| --- | --- | --- | --- | --- | --- | --- | --- | --- | --- | --- |
|  | **Medical schools** | | | | | | | |  | |
| **Variables** | **NTU**  **(*N* = 23)** | **CGU**  **(*N* = 59)** | **KMU**  **(*N* = 86)** | **CMU**  **(*N* = 63)** | **CSMU**  **(*N* = 13)** | **FJCU**  **(*N* = 13)** | **TMU**  **(*N* = 24)** | **NCKU**  **(*N* = 14)** | ***X^2^*** | ***p*** |
| **Gender, male (%)** | 19 (82.6) | 40 (67.8) | 57 (66.3) | 48 (76.2) | 8 (61.5) | 9 (69.2) | 17 (70.8) | 9 (64.3) | 4.277 | .747 |
| **Working hours per week > 80 hours/week** | 4 (25.0) | 5 (19.2) | 1 (1.6) | 5 (13.2) | 5 (55.6) | 3 (33.3) | 0 (0.0) | 0 (0.0) | 32.052 | <.001 |
| **Acceptance of new patients after 24 hours of continuous duty** | 9 (60.0) | 9 (20.5) | 19 (28.4) | 40 (81.6) | 10 (83.3) | 0 (0.0) | 20 (95.2) | 1 (11.1) | 84.820 | <.001 |
| **No 24-hour off within 7 days** | 2 (11.8) | 12 (27.3) | 19 (25.0) | 18 (31.6) | 3 (25.0) | 3 (33.3) | 5 (23.8) | 3 (37.5) | 3.542 | .831 |
|  |  |  |  |  |  |  |  |  | ***F*** | ***p*** |
| **Working hours per week** | 57.31±21.25 | 44.71±29.71 | 44.51±17.17 | 51.58±25.42 | 70.89±39.54 | 68.50±29.84 | 32.63±23.88 | 45.89±5.30 | 3.633 | .001^a^ |
| **Total of self-rating depression score** | 14.00±3.97 | 12.63±3.70 | 12.73±3.77 | 14.44±3.57 | 13.38±4.25 | 12.85 ±2.61 | 14.42±6.37 | 12.86±4.19 | 1.628 | .127 |
| **Score of quality of life** | 51.38±8.38 | 53.26±8.05 | 52.05±6.90 | 49.62±7.27 | 47.48±7.35 | 53.83±8.29 | 47.66±8.80 | 51.07±5.88 | 2.603 | .013^a^ |
| **Score of patient related burnout** | 37.32±10.62 | 32.25±17.37 | 38.78±16.25 | 41.10±14.43 | 34.29±13.84 | 33.33±17.18 | 41.84±20.87 | 34.82±15.21 | 1.933 | .064 |
| Categorical data were summarized as n (%) and continuous data were as mean±SD.  ^a^ All post hoc tests were not significant. | | | | | | | | | | |
