# Supplementary material for: A prospective study of the factors associated with life quality during medical internship
Source: PLoS One. 2019 Aug 12;14(8):e0220608. doi: 10.1371/journal.pone.0220608 (PMC6690540; doi:10.1371/journal.pone.0220608)
Supplement: S2 Table — (DOCX) [file pone.0220608.s002.docx]

**S4 Table.** Effectors of self-rating depression score (*N* = 295 vs. *N* = 55)

| **Variables** | ***N* = 295** | | ***N* = 55** | |
| --- | --- | --- | --- | --- |
|  | **B (95%CI)** | ***p*** | **B (95%CI)** | ***p*** |
| **Specialties of internship rotation** |  |  |  |  |
| Baseline | Reference |  | Reference |  |
| Internal medicine | 0.919 (-0.028,1.866) | 0.057 | 1.203 (-0.259,2.666) | 0.107 |
| Surgery | 1.048 (-0.024,2.119) | 0.056 | 1.013 (-0.660,2.686) | 0.235 |
| Pediatric, obstetrics and gynecology | 0.851 (-0.056,1.758) | 0.066 | 0.717 (-0.733,2.166) | 0.332 |
| Others | 0.953 (0.035,1.872) | **0.042** | 0.433 (-0.905,1.772) | 0.526 |
| **Working hours per week** | 0.013 (0.001,0.025) | **0.041** | 0.008 (-0.006,0.022) | 0.280 |
| **Acceptance of new patients after 24 hours of continuous duty** | 0.904 (0.317,1.480) | **0.003** | 0.954 (-0.094,2.002) | 0.074 |
| **No 24-hour off within 7 days** | 0.828 (-0.019,1.675) | 0.055 | 0.842 (-0.601,2.285) | 0.253 |
| **Score of patient related burnout** | 0.496 (0.385,0.608) | **<.001** | 0.480 (0.295,0.670) | **<.001** |
