# Supplementary material for: A prospective study of the factors associated with life quality during medical internship
Source: PLoS One. 2019 Aug 12;14(8):e0220608. doi: 10.1371/journal.pone.0220608 (PMC6690540; doi:10.1371/journal.pone.0220608)
Supplement: S3 Table — (DOCX) [file pone.0220608.s003.docx]

**S5 Table.** Effectors of WHOQOL-BREF (*N* = 295 vs. *N* = 55)

| **Variables** | ***N* = 295** | | ***N* = 55** | |
| --- | --- | --- | --- | --- |
|  | **B (95%CI)** | ***p*** | **B (95%CI)** | ***p*** |
| **Specialties of internship rotation** |  |  |  |  |
| Baseline | Reference |  | Reference |  |
| Internal medicine | -2.522 (-4.064,-0.981) | **0.001** | -2.433 (-4.408,-0.458) | **0.016** |
| Surgery | -1.909 (-3.578,-0.240) | **0.025** | -1.700 (-3.815,0.414) | 0.115 |
| Pediatric, obstetrics and gynecology | -0.284 (-1.883,1.315) | 0.728 | -0.199(-2.180,1.783) | 0.844 |
| Others | 0.341 (-1.235,1.917) | 0.672 | 0.671 (-1.314,2.657) | 0.507 |
| **Working hours per week** | -0.005 (-0.024,0.014) | 0.627 | -0.002 (-0.024,0.020) | 0.878 |
| **Acceptance of new patients after 24 hours of continuous duty** | -2.089 (-3.140,-1.037) | **<.001** | -1.271 (-2.552,0.009) | 0.052 |
| **No 24-hour off within 7 days** | -1.748 (-2.928,-0.569) | **0.004** | -0.270 (-2.459,1.920) | 0.809 |
| **Score of patient related burnout** | -0.502 (-0.710,-0.294) | **<.001** | -0.725 (-1.020,-0.430) | **<.001** |
| **Total of self-rating depression score** | -1.015 (-1.176,-0.854) | **<.001** | -0.479 (-0.702,-0.255) | **<.001** |
